# Associations between the use of specific psychotropic drugs and all-cause mortality among older adults in Germany: Results of the mortality follow-up of the German National Health Interview and Examination Survey 1998

**DOI:** 10.1371/journal.pone.0210695

**Published:** 2019-01-14

**Authors:** Yong Du, Ingrid-Katharina Wolf, Markus A. Busch, Hildtraud Knopf

**Affiliations:** Department of Epidemiology and Health Monitoring, Robert Koch Institute, Berlin, Germany; University of Malaya, MALAYSIA

## Abstract

**Background:**

Use of psychotropic drugs is common among older adults. Population-based studies on the associations of psychotropic drug use with mortality are sparse.

**Objectives:**

To investigate the associations between the use of specific psychotropic drug groups (opioids, antipsychotics, antidepressants and benzodiazepines) and all-cause mortality among community-dwelling older adults in Germany.

**Methods:**

Participants of the German National Health Interview and Examination Survey 1998 were followed up for mortality from 1997 to 2011. Persons aged 60–79 years with complete data on psychotropic drug use at baseline and on mortality follow-up were considered as study population (N = 1,563). Associations between the use of opioids, antipsychotics, antidepressants and benzodiazepines and all-cause mortality were examined by Cox proportional hazards models adjusted for sociodemographics (sex, age, community size, region, socioeconomic status), life style (smoking, sports, risky alcohol drinking) and health conditions (obesity, disability, history of cardiovascular diseases, diabetes, hyperlipidemia, hypertension, any cancers, any mental disorders) at baseline.

**Results:**

After a median follow-up of 11.4 years, 21, 18, 23 and 26 deaths were documented among those who used at baseline opioids (n = 39), antipsychotics (n = 30), antidepressants (n = 53) and benzodiazepines (n = 54) with an unadjusted mortality rate (MR) of 57.7, 59.1, 44.6 and 53.7 per 1000 person-years, respectively. Meanwhile, 400 deaths were documented among 1,406 nonusers of any of the above mentioned psychotropic drugs with a MR of 26.7 per 1000 person-years. The age and sex adjusted mortality rate ratios in comparison with nonusers were 2.20 (95% confidence intervals 1.42–3.41), 1.66(1.03–2.70), 1.56(1.06–2.28), and 1.57(1.07–2.31) for the use of opioids, antipsychotics, antidepressants and benzodiazepines, respectively. In the fully adjusted Cox models, use of opioids (hazardous ratio 2.04, 95% confidence intervals 1.07–3.89), antipsychotics (2.15, 1.11–4.15) and benzodiazepines (1.76, 1.09–2.82), but not antidepressants, were significantly associated with an increased risk of mortality.

**Conclusions:**

Use of opioids, antipsychotics, benzodiazepines is significantly associated with an increased risk of all-cause mortality among community-dwelling older adults in Germany. Clinicians should be careful in prescribing these psychotropic drugs to older adults while patients already under psychotropic therapy should well balance the risks and benefits of drug use. Further studies with a larger sample size and information on specific indications for psychotropic drug use and mental comorbidities are required to confirm the findings of the present study.

## Introduction

Psychotropic drugs, e.g. antidepressants, anxiolytics and hypnotics, are widely used by older adults for the treatment of neurological and mental health problems such as depression, anxiety, and sleep disorders [1–6]. Older adults who already belong to vulnerable groups (e.g. persons with a worse health status, disability, polypharmacy) are more frequently exposed to psychotropic drug use [7–9]. Safety issues over psychotropic drug use among older adults are a great concern due to comorbidities, polypharmacy as well as changes in pharmacokinetic and pharmacodynamic properties of drugs related to aging processes. Well-documented adverse outcomes of psychotropic drug use among older adults are falls, fractures and injures [10–12], stroke [10, 13], arrhythmia and sudden death [14–16] depending on specific subgroups of psychotropic drugs used.

Mortality related to psychotropic drug use has received much attention in recent years. So far, the association between mortality and the use of specific subgroups of psychotropic drugs (e.g. benzodiazepines and antidepressants) has been investigated among older patients with specific conditions such as dementia, stroke, schizophrenia etc. [12, 17–23]. Results of relevant epidemiological studies have been systematically reviewed [24–30]. Due to wide varieties in study populations, definitions of psychotropic drugs, covariates adjusted, methodological limitations, etc. between studies, findings regarding the risk of death associated with psychotropic drug use have been inconsistent and controversial [24–30]. For example, use of tricyclic antidepressants and of selective serotonin reuptake inhibitors (SSRIs) was found to be associated with an increased risk of all-cause mortality in a large cohort of older patients with diagnosed depression [13], whereas use of tricyclic antidepressants, but not SSRIs was reported to increase mortality among older patients with coronary heart disease who were followed-up for a mean duration of 7.2 years [31]. Use of antipsychotic drugs was found to be associated with a twice high risk of all-cause mortality and little difference exists in risks between use of atypical and typical antipsychotic [29]. In contrast, no evidence of an increased risk of mortality was found for the use of second generation antipsychotics/atypical antipsychotics compared to nonusers [30]. Further, many of previous studies are based on data of patient registers [12] or conducted among patients in institutions such as nursing homes. Important variables on lifestyle or health behaviors that may likely have an influence on mortality are missing in these data sources [12]. Population-representative epidemiological studies with comprehensive collection of data–including data on potential confounders—are needed to assess the association between psychotropic drug use and mortality.

In Germany, one of five community-dwelling older adults has taken at least one psychotropic drug in the past 7 days with opioids, antipsychotics, antidepressants and benzodiazepines being the most frequently used subgroups [7–9]. In the present analysis, we investigated the associations between the use of opioids, antipsychotics, antidepressants and benzodiazepines and all-cause mortality among older adults using data of the mortality follow-up of the German National Health Interview and Examination Survey 1998 (GNHIES98) considering a wide range of important confounders.

## Methods

### Data source: the German National Health Interview and Examination Survey 1997–1999 and its’ mortality follow-up

The German National Health Interview and Examination Survey 1997–1999 (GNHIES98) was conducted by the Robert Koch Institute between October 1997 and March 1999 aiming at providing national population-representative data on the health of adults aged 18–79 years living in Germany. The design, sampling strategy and study protocol have been described in detail previously [32]. The final sample included 7,124 adults (women 3,674, men 3,450) with a response rate of 61.4% [32].

All participants of GNHIES98 who had agreed to be contacted again (N = 6,979, or 98% of GNHIES98 participants) were invited to take part in the National Health Interview and Examination Survey for Adults (DEGS1), which was also conducted by the Robert Koch Institute between November 2008 and November 2011 [33]. DEGS1 used the same sampling methods as GNHIES98 but included more primary sample units(communities), which were randomly sampled from a complete list of German communities proportional to community size. The final sample of DEGS1 included 3,959 former GNHIES98 participants and 4,192 newly recruited persons. The response rate was 42% for the newly recruited participants and 62% for GNHIES98 re-participants [33]. By the end of DEGS1, the vital status of the 6,979 eligible GNHIES98 participants had be obtained and thus completed the mortality follow-up study; 145 (or 2%) GNHIES98 participants who refused to be contacted again missed the study [34, 35]. Of 6,979 GNHIES98 participants, 3,959 persons successfully participated in DEGS1 while 3,020 persons did not. Of 3,959 persons, those (n = 3,045) who complete the interview and examination parts of DEGS1 were censored at the date of examination while those (n = 914) who only completed the interview part of DEGS1 were censored at the date of the phone-interview or the return date of the questionnaire. Those (n = 3,020) who did not participate in DEGS1 were censored at the date of the last inquiry at the local population registry if alive (n = 2,349) or at the date of death (n = 671, women 285, men 386) [34, 35]. The 6,979 GNHIES98 participants were followed-up for a median of 12 years with a total of 80,742.5 person-years [34].

Both GNHIES98 and DEGS1 conform to the principles outlined in the Declaration of Helsinki and to the German Federal Data Protection Act. Study protocols were approved by the Federal and State Commissioners for Data Protection. The mortality follow-up study is a component of DEGS1, which was approved by the Charité-Universitätsmedizin Berlin ethics committee (No. EA2/047/08). All survey participants provided a written informed consent prior to interview and examination.

### Data collection, definition of study variables and study population

All survey participants at baseline answered a standardized, self-administered questionnaire on sociodemographic characteristics, medical history, health-related behavior such as smoking status, alcohol consumption, or sports activities. A standardized face-to-face, computer-assisted personal interview (CAPI) was conducted by trained physicians and a detailed medical history of pre-existing, physician-diagnosed chronic health problems such as cardiovascular disease (CVD), hypertension, hyperlipidemia, diabetes, any cancer etc. was obtained [32]. A total of 44 chronic health problems were listed in CAPI. For chronic health problems with significant public health impact such as hypertension, CVD, diabetes, asthma and allergic diseases, we further collected disease-specific data.

#### -Psychotropic drug use

Data of medication use at baseline were also documented during the medical interview. In the invitation letter, participants were asked to bring the original packages of all medicines and dietary supplements used during the past seven days–prescribed and Over-The-Counter (OTC) products- to the examination site for the purpose of documentation and drug use verification. Definition of psychotropic drugs has been published in our previous analyses [7, 9]. In brief, we included all drugs belonging to the nervous system class (ATC code N) excluding analgesics and antipyretics under the ATC code N02B (e.g. aspirin and paracetamol), as well as local anesthetics (ATC code N01B), homeopathic drugs, and drugs with indistinct active ingredients, but retaining aspirin-caffeine combination preparations (ATC code N02BA71) in the analysis [7, 9]. In the presents study we considered only the use of specific subgroups of psychotropic drugs, namely opioids used as analgesics (N02A) or antitussives (R05DA), antipsychotics (N05A), antidepressants (N06A) and benzodiazepines (N05BA, N05CD, N03AE) as they were the most frequently used psychotropic drugs among older adults with a relatively large number of users compared to other subgroups [7]. Opioids acting on opioid receptors yield both psychoactive/euphoric and somatic effects. Given its potential abuse and increased number of deaths involving opioids [36–38], we included opioids in the present study. Nonusers of psychotropic drugs were defined as those with no use of any above mentioned psychotropic drugs.

For each drug we recorded, we collected data on duration of drug use by asking survey participants “how long has the drug been used?”. Possible answerer choices were 8 categories (from “less than 1 week”, “1 week”, to “6 months-1 year”, “1–3 years” and “over 3 years”). This information was grouped into three categories as short-term (<1 year), medium-term (1–3 years) and long-term use (> = 3 years).

#### -Study population

In the presents study, we included only participants who were 60 years of age or older with complete data on the use of the here included psychotropic drugs at baseline (GNHIES98) and information on vital status at the follow-up (November 2011). Thus, the study population amounted to 1,563 (women 854, men 709) adults aged 60–79 years at baseline (Table 1).

**Table 1:** Descriptive characteristics of study population at baseline. Comparison between psychotropic drug users and nonusers. GNHIES98 mortality follow-up study.

	Opioids (N02A& R05DA)	Antipsychotics (N05A)	Anti-depressants (N06A)	Benzodiazepines (N05BA, N05CD, N03AE)	Nonusers
Total, *N*	39	30	53	54	1406
Mean age (SE), *yrs*	69.7(.9)*	70.8 (.9)**	69.6 (.9)*	69.9(.8)*	67.7 (.2)
Women, %	65.6	61.9	76.0*	65.6	55.7
West Germany, %	79.1	71.6	84.0	78.4	77.4
Residing in cities, %	71.7	57.6	56.1	69.5	61.2
Social status, %					
*Lower*	28.4	30.3	38.0	23.4	25.5
*Middle*	67.4	60.4	52.3	58	60.2
*Upper*	4.2	9.2	9.7	18.6	14.3
Current smoking, %	20.0	11.6	10.0	15.8	14.0
No sports at all, %	80.8	76.8	70.3	63.6	67.3
Risky drinking, %	7.2	3.2*	12.5	17.8	16.9
Certified disability, %	42.6*	32.1	24.5	38.6	24.7
Chronic conditions, %					
*Obesity*	35.2	22.5	30.3	31.3	30.2
*CVD*	30.0	22.8	42.1*	43.6*	26.9
*Diabetes*	10.8	13.8	21.1	13.8	14.1
*Hyperlipidemia*	45.7	21.9*	37.2	44.0	41.0
*Hypertension*	58.0	47.5	52.3	52.7	47.7
*Any cancer*	14.3	9.1	8.7	11.1	7.5
*Any mental health disorder*	20.8*	56.3**	60.0**	41.3**	7.8
Duration of use, %					
*Long-term users (> = 3 years)*	32.7	43.0	56.8	62.1	-
*Medium-term users (1–3 years)*	32.4	22.3	22.5	21.0	-
*Short-term users < 1 year)*	35.0	34.7	20.7	16.9	-

All percentages and means were weighted and standardized to the population of 31.12.1997

Cities: population >20.000 residents

Risky drinking: average daily consumption of alcohol ≥10 g for women, and ≥20 g for men

CVD: cardiovascular diseases (including coronary heart diseases, heart failure, and stroke)

Obesity: defined as body mass index> = 30 kg/m^2^

Nonuser: no use of any psychotropic drugs (opioids, antipsychotics, antidepressants and benzodiazepines)

* P < .05

** p < .001, chi-square test, compared with nonusers.

#### -Co-variables

Co-variables that are likely to be associated with mortality and considered in the present analysis included socio-demographic characteristics (age, sex, residing region, community size), individual socio-economic status, health behaviors (smoking, sports, risky alcohol drinking), and health conditions (obesity, disability, medical history of chronic diseases) at baseline.

Residing region by federal states and community size by population density were defined as described previously [7–9, 39]. Sports activity in the past three months was assessed by five categories (no sports, <1 hour/week, regularly 1–2 hours/week, regularly 2–4 hours/week, regularly >4 hours/week). Smoking status was assessed as never, former and current smoking. Considering sample size and distribution of each variable in the study population, all these co-variables were dichotomized (Table 1). Alcohol consumption (grams per day) was determined according to the type and volume of alcohol-containing beverages [7–9, 39]. In Germany, the threshold for risky alcohol drinking is set at ≥ 10–12 grams/day of alcohol for women and ≥ 20–24 grams/day for men. As in our previous studies, we adopted the lower limits of ≥ 10 grams/day for women and ≥ 20 grams/day for men to define risky drinking [7–9, 39]. Individual socio-economic status (SES) was classified as ‘lower’, ‘middle’ and ‘upper’ using an established index including information on education, professional status and household income [40]. Survey participants were asked if they had an officially certified disability (yes/no) by self-administered questionnaires. Obesity was defined as body mass index (BMI) > = 30 kg/m^2^, which was calculated as the ratio of measured body weight (kg) and body height (meters) squared. Information on history of chronic diseases such as CVD (including coronary heart diseases, heart failure, stroke), hypertension, hyperlipidemia, diabetes, any cancers and any mental health disorder was obtained during the CAPI by trained physicians with the question “Has a doctor ever told you that you have the following disease?”. There was no clear definition for mental health disorder except that 3 examples were given (anxiety, depression, and psychosis). Other mental health related chronic problems including cognitive impairment or dementia, addiction to medications, alcohol and other substances, abnormal eating disorders as well as Parkinson disease were measured independently as one of the 44 listed chronic health problems in CAPI and thus were not included in the above mentioned mental health disorder.

### Statistical analysis

All statistical analyses were performed using Stata (version 14.0, StataCorp, College Station, TX, USA). A weighting factor was computed to adjust for deviations in demographic characteristics (age, sex, education, nationality, federal state, and level of urbanity) between the study population and official population statistics of 31.12.1997.

Descriptive statistics were used to examine characteristics of study population at baseline with comparisons between psychotropic drug users and nonusers. Crude mortality rate (MR) and 95% confidence intervals (CIs) for psychotropic drug users and nonusers were calculated by the jackknife method. Age and sex adjusted mortality rate ratios (MRR) and 95% CIs comparing MR of psychotropic drug users and nonusers were calculated by using Mantel-Haenszel estimates. Hazard ratios (HR) and 95% CIs for mortality were derived from Cox proportional hazards regression models adjusted for the co-variables described above. Follow-up time, as the dependent time variable, was computed as time interval between the date of baseline examination in GNHIES98 and the date of re-contact if alive or the date of death if deceased. Considering that overall 6% of study subjects have missing values in some variables (ranging from 1.1% for BMI, 3.7% for smoking and social status, 4.0% for sports and certified disability, to 4.2% for risky drinking), we used multiple imputations by chained equations for missing values in the regression analyses assuming missing at random [41]. For each Cox regression model, 15 imputed datasets were created. We performed sensitivity analyses by repeating all regression analyses with a complete-case dataset (i.e. including only observations with complete information for all covariables in the Cox model) and compared estimates with those obtained by using multiple imputated datasets. Statistical significance was defined at p<0.05 based on two-sided tests.

## Results

Table 1 shows the baseline descriptive characteristics of the study population. Among the study population, a total of 39, 30, 53 and 54 persons used opioids, antipsychotics, antidepressants and benzodiazepines, respectively, at baseline. The most frequently observed combination use was antidepressants + antipsychotics (n = 8), followed by antidepressants + benzodiazepines (n = 7) and opioids + benzodiazepines (n = 3) (data not shown in the Table). Compared to psychotropic drug non-users, users in each group of psychotropic drugs were on average 2–3 years older and, as expected, had a higher prevalence of mental disorders (Table 1). In addition, benzodiazepine users were more likely to have a CVD while antipsychotics users were less likely to have hyperlipidemia or to drink riskily. More antidepressants users were women and they had a higher prevalence of CVD. Opioid users had a higher proportion with certified disability (Table 1). The majority of psychotropic drugs have been used over 1 year at baseline; the proportion of long-term users (over 3 years) was 32.7% for opioids, 43.0% for antipsychotics, 56.8% for antidepressants and 62.1% for benzodiazepines (Table 1).

Table 2 shows the weighted crude MRs for psychotropic drug users and non-users as well as MRR for psychotropic drug users vs. non-users. The study population was followed-up for a mean duration of 11.4 years (range 0.05–14.1 years). During the follow-up, 21, 18, 23 and 26 deaths were documented in the users of opioids, antipsychotics, antidepressants and benzodiazepines, with an unadjusted MR of 57.7, 59.1, 44.6 and 53.7 per 1000 person-years, respectively (Table 1). Meanwhile, 400 deaths were documented among 1,406 nonusers with an unadjusted MR of 26.7 per 1000 person-years (Table 1). The age and sex adjusted MRR was 2.2, 1.66, 1.56, and 1.57 for the use of opioids, antipsychotics, antidepressants and benzodiazepines, respectively, in comparison to nonusers (all statically significant).

**Table 2:** Mortality rates and mortality rate ratios by the use of psychotropic drugs. GNHIES98 mortality follow-up study.

	Total (N)	No. of deaths (n)	Follow-up (person-years, PY)^1^	Mortality rates (per 1000 PY)^1^	95% CI^1^	Mortality rate ratios (MRR)^1^^,^^2^	95% CI^1^^,^^2^	p
Psychotropic drug users (ATC-code)								
Opioids (N02A & R05DA)	39	21	379.5	57.7	33.1–101.9	2.20	1.42–3.41	**.000**
Antipsychotics (N05A)	30	18	300.9	59.1	36.5–97.4	1.66	1.03–2.70	**.037**
Antidepressants (N06A)	53	23	674.8	44.6	30.4–67.6	1.56	1.06–2.28	**.022**
Benzodiazepines (N05BA, N05CD, N03AE)	54	26	550.8	53.7	36.9–79.6	1.57	1.07–2.31	**.020**
No use of any above drugs	1406	400	15454.6	26.7	23.9–29.8	1.00 (ref.)		

^1^ Weighted and standardized to the population of 31.12.1997

^2^ Age and sex adjusted

The vast majority of antidepressants were Non-Selective Monoamine Reuptake Inhibitors (NSMRIs, ATC code N06AA) (n = 45), only 2 users of Selective Serotonin Reuptake Inhibitors (SSRIs, ATC code N06AB) were recorded at baseline. 21 deaths among NSMRIs users and 1 death among SSRI users were documented in the follow-up. The age and sex adjusted MRR was 1.58 (95% CI 1.06–2.38) and 2.52 (0.68–9.37) for the use of NSMRIs and SSRIs, respectively (Data not shown in Table 2). 12 persons (40% of antipsychotic users) were users of atypical antipsychotics with 6 deaths being recorded in the follow-up. The age and sex adjusted MRR was 1.22 (0.60–2.49) for the use of atypical antipsychotics and 2.37 (1.25–4.52) for the use of typical antipsychotics, respectively (Data not shown in Table 2).

Table 3 depicts mortality hazard ratios (HR) for the use of psychotropic drugs. After adjusting for age, sex, social status, residing in cities and West Germany, smoking, sports, risky drinking, obesity, recognized disability, history of hypertension, hyperlipidemia, cardiovascular diseases, diabetes, any cancers and any mental disorders, use of opioids, antipsychotics, benzodiazepines, but not antidepressants, was significantly associated with an increased risk of mortality (Table 3). Further looking at the subgroup of antidepressants revealed use of SSRIs (HR 8.81, 4.02–19.3, based on n = 2 SSRIs users and 1 death), but not NSMRIs (HR 1.20, 0.66–2.18), was associated with an increased risk of mortality (data not shown in Table 3). Neither use of atypical antipsychotics (HR 1.89, 0.87–4.87) nor use of typical antipsychotics (HR 2.57, 0.81–8.14) was significantly associated with an increased risk of mortality (data not shown in Table 3).

**Table 3:** All-cause mortality hazard ratio (HR) for the use of psychotropic drugs. GNHIES98 mortality follow-up study.

Psychotropic drug users (ATC-code)	HR^1^	95% CI^1^	p
Opioids (N02A & R05DA)	2.11	1.17–3.82	**.013**
Antipsychotics (N05A)	2.35	1.31–4.23	**.005**
Antidepressants (N06A)	1.44	0.87–2.39	.155
Benzodiazepines (N05BA, N05CD, N03AE)	1.79	1.01–3.19	**.047**

^1^ Weighted and standardized to the population of 31.12.1997

Hazard ratio (HR) and 95% confidence intervals (95% CI) were derived from Cox proportional hazards models, adjusted for sex and age (continuous), social status (lower, middle, upper), residing in cities, West Germany, obesity, current smoking, no sports, risky alcohol drinking, recognized disability, history of hypertension, hyperlipidemia, cardiovascular diseases, diabetes, any cancer and any mental disorders (all yes vs. no).

Reference: Nonusers (no use of any above listed psychotropic drugs)

Stratified by duration of drug use, no difference was found for the use of all psychotropic drugs > = 3 vs. <3 years.

Sensitivity analysis by using complete case methods found little difference in comparison with results in Table 3: HR 2.04 (1.07–3.89) for opioids, HR 2.15 (1.11–4.15) for antipsychotics, HR 1.24 (0.70–2.19) for antidepressants and HR 1.76 (1.09–2.82) for benzodiazepines use. Further adjusting for physician-diagnosed dementia or excluding combination use of psychotropic drugs for each group revealed no substantial changes.

**Fig 1:**
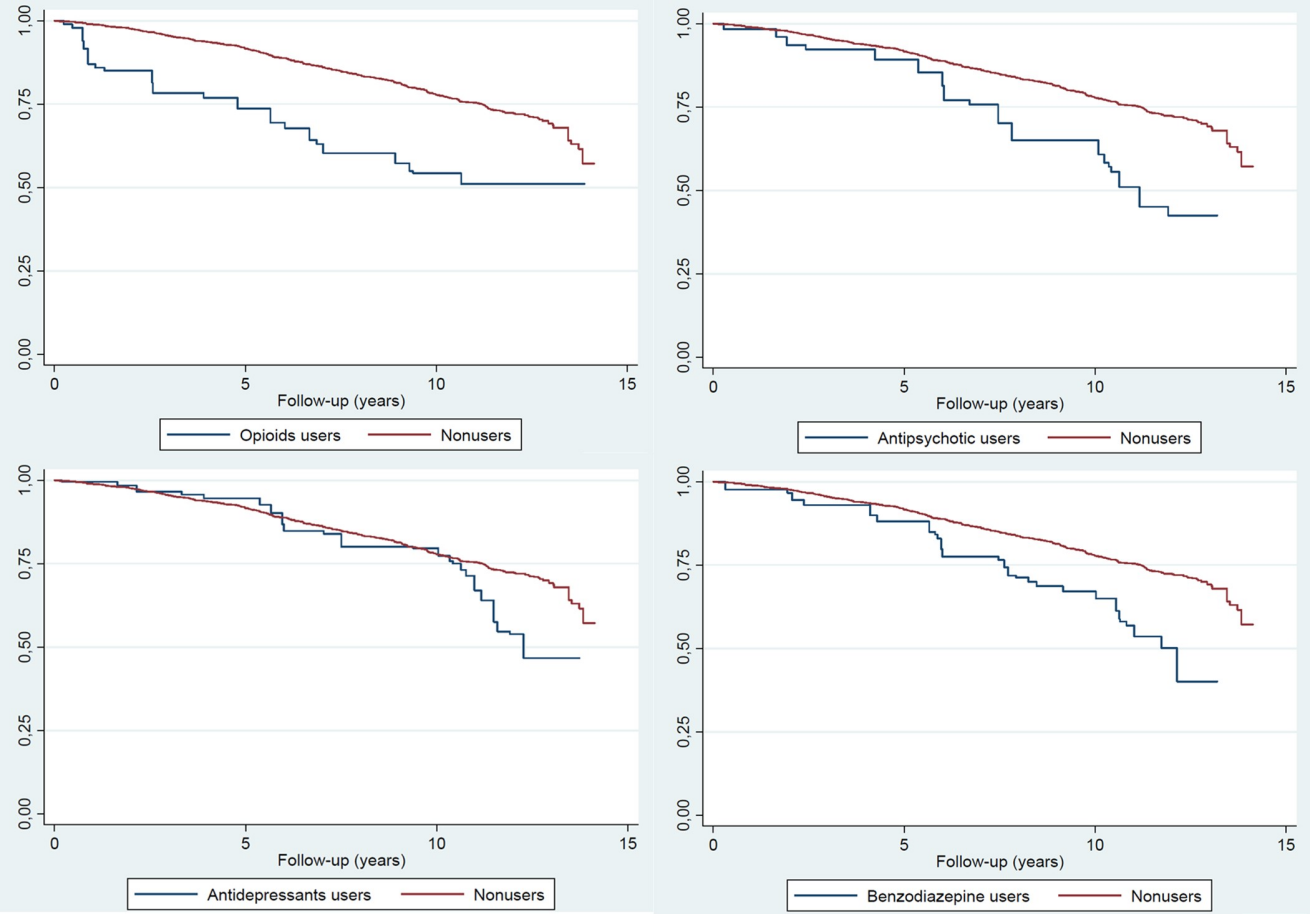
Kaplan-Meier survival estimates for the use of opioids, antipsychotics, antidepressants and benzodiazepines in comparison with nonusers (nonusers: No use of any opioids, antipsychotics, antidepressants and benzodiazepines). GNHIES98 mortality follow-up study.

Kaplan–Meier survival estimates for all-cause mortality stratified by psychotropic drug uses are shown in Fig 1. Along with the follow-up years, the survival probability decreased both for psychotropic drug users and nonusers. Nevertheless, the survival probability decreased faster for users of opioids, antipsychotics and benzodiazepines, but not for users of antidepressants, than for nonusers.

## Discussion

### Main findings

In the present analysis, we found that use of opioids, antipsychotics and benzodiazepines was associated with an increased risk of mortality among community-dwelling older adults after a mean follow-up of 12 years. Associations of an increased risk of mortality with the use of antidepressants were found to be significant only in the age and sex adjusted models, but not in the fully adjusted Cox models.

### Comparison with other studies

Comparing our results with findings of other studies might be difficult as there are great varieties between studies concerning study population, data collection, adjustment for confounders, and in particular, definition of psychotropic drugs. In spite of this, an increased risk of mortality following the use of psychotropic drugs in older people has been reported in many epidemiological studies. For example, registry-based studies have examined the association of psychotropic drug use with all-cause mortality among patients with a specific disease and among matched controls without the disease [17, 22, 42]. Among matched controls without Parkinson disease, a significantly higher risk of all-cause mortality was found for the use of SSRIs or serotonin-noradrenalin reuptake inhibitors (HR 1.77; 95% CI 1.64–1.91), benzodiazepines (1.39; 1.29–1.51), first-generation (2.12; 1.82–2.47) and second-generation (2.00; 1.66–2.43) antipsychotics [42]. Similar results are also consistently found in controls without dementia [22] or stroke [17] and also among patients with this disease [17, 22, 42]. Another large register-based case-control study reported that use of antipsychotics, anxiolytics, antidepressants in the past year was strongly related to death with an odds ratio of 2.10 (95% CI 2.02–2.17), 1.35 (1.32–1.38) and 1.43 (1.40–1.46), respectively [12].

In our study, we found that the use of antidepressants was associated with an elevated risk of mortality in age and sex adjusted model, but not in the fully-adjusted Cox model. Although we found SSRIs increased the risk of mortality, this was based only on 2 SSRIs users and 1 death case. Use of antidepressants has been linked with sudden death [43] and suicide [44, 45]. Among patients with depression treated with antidepressants, it remains, however, controversial whether increased risk of suicide is due to the medications used or the depression itself. Pharmacologically treated depression has been associated with a 49% increased mortality risk (HR 1.49; 1.03–2.16) among patients with systolic heart failure [46], which was confirmed by a followed-up study on older outpatients with heart failure [47]. Within the subgroup of antidepressants, different effects of SSRIs and tricyclic antidepressants on mortality were reported. While the use of any antidepressants (HR 0.89; 0.71–1.13) and SSRIs (0.90; 0.71–1.15) was not independently associated with mortality, use of Fluoxetine, a SSRI, was associated with increased mortality (HR 1.66; 1.13–2.44) independent of depression symptoms [47]. Zivin et al assessed the hazards of 1-year all-cause mortality associated with antidepressants use by utilizing conventional and propensity-stratified Cox regression, and 2 forms of marginal structural models [48]. The authors concluded that antidepressants use was associated with no excess harm when accounting for clinical and demographic characteristics and treatment selection bias [48], similar to our findings.

Different from analgesics such as aspirin, ibuprofen etc., narcotic analgesics act on opioid receptors producing morphine-like effects, which are mainly used for relief of cancer pain because of their high potential of addiction. However, they are also increasingly used in the treatment of non-cancer pain, which is paralleled by an increase in overdose deaths [49]. In a prospective cohort study of 50,045 participants aged 40–75 who were enrolled from 2004 to 2008 and followed up for a mean duration of 12.7 year, Khademi and colleagues found that opium use is associated with almost double the risk of death from any cause (HR 1.86, 1.68 to 2.06) [50]. Opium consumption increased risks of deaths across a variety of diseases including cardiovascular disease, cancers [50], but also respiratory diseases [51] and digestive disease [52]. As with opium, the use of opioids may also increase the risk of all-cause mortality. We found that use of opioids was associated with a double risk of death among older adults in Germany. In a propensity score matched cohort study conducted among older adults with osteoarthritis in the United States, it was found that use of prescribed opioids raised the risk of all-cause mortality (HR, 1.87; 1.39–2.53) compared with the use of non-steroidal anti-inflammatory drugs [53]. A more recent US propensity score-matched retrospective cohort study comparing risks of mortality between users of long-acting opioids and controls reported a significantly higher mortality risk among users of opioids in comparison to users of analgesic anticonvulsants or low-dose cyclic antidepressants (HR 1.64, 1.26–2.12) [49].

In the clinical practice, benzodiazepines are mainly used as sedatives, hypnotics, anxiolytics and anticonvulsants. In a systematic review [27], a significantly higher mortality risk was found among benzodiazepine users vs. non-users (HR 1.60; 1.03–2.49) [27], which is supported by another review including publications of the past 30 years [54]. However, in a population-based retrospective cohort study [55] use of benzodiazepines and benzodiazepine-related drugs was found to be associated with an increased mortality hazard in unadjusted analyses (HR 1.53; 1.28 to 1.82), but not in fully adjusted analysis (HR 1.01, 0.84 to 1.21). Health behaviors and health conditions could explain the most part of effects as the principal confounders of the observed associations in unadjusted models [56, 57]. Results of register-based studies have adjusted for a number of covariates including comorbidities, but not lifestyle factors [12, 17, 22, 42]. These results suggest that adjusting for essential co-variables is needed and may play a critical role in studies on association of psychotropic drug use with mortality.

We found that use of antipsychotic drugs overall was associated with a twice high risk of mortality. This is consistent with findings of a recently published meta-analysis [29]. Based on data of studies conducted between 2009–2017 including from over 380,000 dementia patients as well as from 359,235 non-dementia antipsychotic drug users, the authors reported a pooled HR of 1.934 (1.723–2.171) with little difference between using typical and atypical antipsychotic drugs [29]. Yet, in another review including only randomized clinical trials, no evidence was found that the second generation antipsychotic drugs increase mortality [30]. In our study, a nonsignificant HR with wide confidence intervals for the use of both typical and atypical antipsychotic drugs in the fully adjusted model does not mean that it is not associated with an increased risk of mortality due to small sample size by stratified analyses.

### Strengths and limitations

Our study has several strengths. First, we used a nationally representative sample of an older adult population in Germany. Vital status was confirmed by 98% of participants of the national health survey after a mean follow-up of 12 years. The weighted results can be generalized to the German community-dwelling older adults aged 60–79 years. Second, we fitted Cox regression models to explore the associations between psychotropic drug use and mortality controlling for a number of confounding variables including life styles and chronic health conditions.

Results of our study are subject to several limitations, however. First, our results are not representative of the entire older population in Germany. Persons aged 80 years and older or persons hospitalized or institutionalized (e.g. those in nursing-homes) were pre-excluded in the national health survey. Further, community-dwelling older adults with cognitive impairment, depression or other severe mental or physical illnesses might be less likely to take part in the baseline survey. These persons are expected to have a higher psychotropic drug use and a higher probability of mortality. Including these persons in our study would have probably strengthened our findings. Second, information on drug use, health behavior and health conditions during the period of follow-up were unavailable. The influence of these factors on mortality was impossible to be considered in the present analysis. For example, some psychotropic drugs used at baseline are not necessarily continued to be used for many years. In the present study, the majority of psychotropic drugs have been used over 1 year and the proportion of long-term psychotropic drug users (over 3 years) was high (Table 1). Third, the limited number of psychotropic drug users, particularly in subgroups and of mortality cases, lowered the statistical power, preventing us from detecting weak associations. Generally, it is impossible for us to do any stratified analysis such as by use duration for a possible dose-response relationship or by type of mortality for any cardiac events/strokes related risks. These stratified analyses are of clinical relevance and should be explored further in epidemiological studies with large sample size. Fourth, the GNHIES98 was conducted in the late of 1990s. Since then, clinical practice in the use of psychotropic drugs—antidepressants and antipsychotics in particular—has been changed. For example, SSRIs and atypical antipsychotic drugs are increasingly used. Risk of mortality for use of these drugs among older adults should be further investigated. Finally and most importantly, as with all observational findings, our results suggest the association of baseline drug use with mortality only and are subject to indication bias. Indication bias occurs when patients are prescribed drugs for a condition that itself is associated with the outcome of interest. Although we have adjusted for many factors including mental health disorders, systematic differences are still likely between patients who are treated with drugs and those who are not. Persons already under psychotropic therapy with opioids, antipsychotics, antidepressants, etc. might be at an advanced stage of disease and thus have a higher risk of mortality.

### Conclusions

In summary, in the mortality follow-up study of the German national health survey 1998, we found that the use of opioids, antipsychotics and benzodiazepines was associated with an increased risk of all-cause mortality among community-dwelling older adults in spite of overall small sample size. As with all other observational studies, our results should be interpreted with prudence given limitations such as small sample sizes, indications bias, considered confounders and significant differences between psychotropic drug users and nonusers. Further studies with a larger sample size and information on specific indications for psychotropic drug use and mental comorbidities are required to confirm the findings of the present study. In spite of this, supported by results of previous epidemiological studies, the main findings of our study added evidence that use of these specific psychotropic drugs increases all-cause mortality among older adults. Clinicians should be cautious in prescribing these psychotropic drugs to older adults and patients already under psychotropic therapy should balance the risks and benefits of drug use.
